# Bilateral adrenal adenomas presenting with ACTH-independent Cushing’s syndrome and primary aldosteronism: diagnostic insights from multi-steroid adrenal venous sampling

**DOI:** 10.3389/fendo.2026.1861198

**Published:** 2026-08-03

**Authors:** Rong Long, Yu Li, Xinyi Gong, Run Wu, Qinfei Yang, Dejun Wang, Ying Yang, Nan Chen

**Affiliations:** 1 Department of Endocrinology, Anning First People’s Hospital Affiliated of Kunming University of Science and Technology, Kunming, Yunnan, China; 2 Department of Radiology, Anning First People’s Hospital Affiliated of Kunming University of Science and Technology, Kunming, Yunnan, China; 3 Department of Laboratory, Anning First People’s Hospital Affiliated of Kunming University of Science and Technology, Kunming, Yunnan, China; 4 Department of Endocrinology, Affiliated Hospital of Yunnan University, Kunming, Yunnan, China

**Keywords:** adrenal venous sampling, adrenocorticotropic hormone-independent Cushing’s syndrome, aldosterone-and cortisol-co-secreting adenomas, bilateral adrenal, primary aldosteronism

## Abstract

**Background:**

Primary aldosteronism (PA) is the most prevalent cause of secondary hypertension (HTN), and adrenocorticotropic hormone (ACTH)-independent Cushing’s syndrome (CS) represents a distinct form of adrenal-derived secondary HTN. Their coexistence resulting from aldosterone- and cortisol-producing adenomas (A/CPAs) is an extremely rare clinical entity. This is particularly true for the subtype characterized by bilateral adrenocortical adenomas, in which one adenoma predominantly secretes aldosterone (ALD) and the other predominantly secretes cortisol (COR), for which only limited clinical reports have been published to date.

**Case description:**

Both female patients were diagnosed with PA and concurrent ACTH-independent CS. Abdominal computed tomography (CT) revealed bilateral adrenal space-occupying lesions. Adrenal venous sampling (AVS) was subsequently performed using 17α-hydroxyprogesterone (17-OHP), androstenedione (ASD), dehydroepiandrosterone (DHEA) as the reference hormone, which demonstrated lateralized dominance of COR and ALD secretion from opposite adrenal glands. Corresponding clinical features confirmed the diagnosis of A/CPAs in both cases. One patient underwent laparoscopic left adrenal CPA resection, while the other underwent laparoscopic right adrenal CPA resection. Following laparoscopic resection of the CPA in both patients, postoperative COR levels declined, consistent with surgical remission. Spironolactone therapy was initiated. Pathological examination confirmed adrenocortical adenoma. Immunohistochemistry (IHC) showed strong positive expression of CYP11B1 (11β-hydroxylase) and weak expression of CYP11B2 (ALD synthase). Their conditions have remained well controlled to date.

**Conclusions:**

In patients with bilateral adrenal adenomas and concurrent PA and ACTH-independent COR excess, imaging alone may fail to identify the functional source of hormone excess. Multi-steroid adrenal venous sampling demonstrated opposing ALD and COR dominance from contralateral adrenal glands, guiding targeted resection of the COR-dominant lesion and postoperative medical control of residual ALD excess. These cases support multi-steroid AVS for individualized adrenal-sparing management.

## 1 Introduction

Primary aldosteronism (PA), affecting an estimated 5%–10% of individuals with hypertension, is the most common cause of secondary hypertension (HTN) (1).PA is characterized by autonomous or semi-autonomous aldosterone (ALD) overproduction from one or both adrenal glands. Excess ALD suppresses the renin–angiotensin system, promotes renal sodium retention and intravascular volume expansion, and may cause hypokalemia in more severe cases (2). PA is broadly classified into two major subtypes: unilateral PA, most commonly caused by aldosterone-producing adenoma (APA) or unilateral adrenal hyperplasia, and bilateral PA, characterized by bilateral ALD overproduction (3, 4). Adrenocorticotropic hormone (ACTH)-independent Cushing’s syndrome (CS) is another adrenal-derived cause of secondary HTN, with cortisol (COR)-producing adenomas being a major etiology (5, 6).Autonomous COR secretion causes ACTH-independent hypercortisolism and suppress hypothalamic–pituitary ACTH secretion through negative feedback (7). Even in the absence of overt Cushingoid features, mild autonomous COR secretion is associated with cardiometabolic complications, including difficult-to-control HTN and impaired glucose metabolism (8).

An aldosterone-and cortisol-producing adenoma (A/CPA) is typically a unilateral and solitary adrenal adenoma that autonomously co-secretes ALD and COR, leading to PA with concomitant autonomous COR secretion; its clinical spectrum ranges from mild autonomous cortisol secretion to overt ACTH-independent CS (9, 10). To date, reports of A/CPAs remain scarce, and most documented cases have involved either a single adenoma co-secreting both hormones or ipsilateral double adenomas (9, 11).In contrast, the two patients described in this report had bilateral adrenal lesion with opposing hormonal dominance: one adrenal adenoma was predominantly responsible for COR excess, whereas the contralateral lesion was predominantly responsible for ALD excess. This rare “opposing secretion” pattern has important clinical implications. First, cross-sectional imaging cannot reliably determine the functional source of each hormone. Second, in the presence of autonomous COR secretion, conventional COR-corrected ALD ratios may be misleading; therefore, multi-steroid adrenal venous sampling (AVS) using non-COR reference steroids may be particularly useful for accurate functional lateralization and surgical decision-making. In addition, resection of a COR-secreting adrenal tumor may leave the contralateral adrenal gland suppressed, thereby complicating postoperative glucocorticoid management and potentially necessitating temporary steroid replacement. Therefore, concomitant autonomous COR secretion should be carefully evaluated in patients with PA (12). However, the diagnostic and therapeutic challenges posed by this “opposing secretion” pattern, particularly in AVS interpretation and surgical planning, remain insufficiently addressed. Here, we report two such cases to highlight this distinctive entity and its clinical significance.

## 2 Case presentation

### 2.1 Case 1

A 43-year-old premenopausal woman was referred to our endocrinology department for evaluation of refractory HTN and incidentally discovered bilateral adrenal lesions. Three years prior, she had presented with severe HTN (206/110 mmHg) and was sequentially managed with indapamide, amlodipine, and irbesartan–hydrochlorothiazide, yet her blood pressure remained labile (130–160/90–100 mmHg). One month prior to admission, the patient was admitted elsewhere for headache and was found to have recurrent hypokalemia during hospitalization; however, records from the referring institution were unavailable. Non-contrast abdominal CT revealed a nodular slightly low hypodense lesion in the body of the right adrenal gland and an oval isodense lesion in the left adrenal region. Following consultation with our endocrinology department, her antihypertensive regimen was adjusted to include verapamil hydrochloride 120 mg three times daily, and she was admitted to our unit four weeks later. She had a 6-month history of type 2 diabetes mellitus (T2DM), managed with metformin sustained-release tablets 0.5 g three times daily. She underwent cholecystectomy 9 years prior. There was no history of coronary artery disease, infectious diseases, smoking, or alcohol consumption. She was unmarried, nulliparous, and had regular menstrual cycles. Family history was notable only for maternal HTN, with no reported family history of diabetes. On admission, vital signs revealed stage 2 HTN (161/100 mmHg) and a regular heart rate of 82 bpm. Anthropometric assessment demonstrated a BMI of 22.15 kg/m², waist circumference of 92 cm, and a waist-to-hip ratio (WHR) of 1.03. Notably, there was no clinical evidence of hypercortisolism, including central obesity, moon facies, buffalo hump, or striae. Cardiopulmonary and abdominal examinations were otherwise unremarkable, with preserved muscle strength and tone in all extremities.

Baseline metabolic evaluation revealed an glycated hemoglobin (HbA1c) of 7.3%, fasting plasma glucose 5.65 mmol/L, fasting serum insulin 12.6 μU/mL, and C-peptide 4.73 ng/mL. alongside hyperuricemia (465 μmol/L). Lipid panel showed triglycerides 1.58 mmol/L, total cholesterol 3.17 mmol/L, high-density lipoprotein cholesterol 1.76 mmol/L, and low-density lipoprotein cholesterol 1.09 mmol/L. Serum potassium was 3.77 mmol/L, accompanied by 24-hour urinary potassium 37.06 mmol and 24-hour urinary sodium 169.38 mmol. Upright direct renin concentration (DRC) was 3.02 pg/mL, upright plasma ALD 189.11 pg/mL, resulting in an upright aldosterone-to-renin ratio (ARR) of 62.62. Saline infusion testing was performed (**Table 1**). Furthermore, COR circadian rhythm was assessed (**Table 2**). These findings established the diagnosis of ACTH-independent CS. Plasma-free and urinary metanephrines (MNs) were within normal limits.

**Table 1:** Interpretation of laboratory tests for PA.

	Screening			Confirmation				Subtyping	
Case	Upringt			Saline infusion test		Captopril challenge test		1 mg DST suppression test combined with ACTH stimulation test	
	DRC(pg/ml)	ALD(pg/ml)	ARR	Pre-infusion ALD(pg/ml)	Post-infusion ALD(pg/ml)	Pre-drug 0h ALD(pg/ml)	Post-drug 2h ALD(pg/ml)	Pre-infusion 0min ALD (pg/ml)	Post-infusion 90min ALD (pg/ml)
1	3.02	189.11	62.62	263.12	175.59	223.17	168.59	189.72	341.42
2	2.41	194.22	80.59	240.48	100.26	141.73	119.30	195.98	381.43
Reference range	3.8-38.8	40-310	<38	40-310					

PA, primary aldosteronism; ALD, aldosterone; DRC, direct renin concentration; ARR, aldosterone-to-renin ratio; ACTH, adrenocorticotropic hormone; DST, Dexamethasone.

**Table 2:** CS screening and confirmation.

	COR circadian rhythm									
Case	08:00		16:00		00:00		1mg DST		LDDST	
	ACTH (pg/ml)	COR (ug/dl)	ACTH (pg/ml)	COR (ug/dl)	ACTH (pg/ml)	COR (ug/dl)	ACTH (pg/ml)	COR (ug/dl)	ACTH (pg/ml)	COR (ug/dl)
1	<1.00	26.51	<1.00	21.36	<1.00	20.36	<1.00	26.84	2.62	27.03
2	1.19	10.63	1.19	10.13	1.26	8.84	—	10.95	8.56	12.58
Reference range	7.2-63.4	4.26-24.85	7.2-63.4	2.9-17.3	7.2-63.4	<1.8	—	<1.8	—	<1.8

CS, Cushing,s syndrome; ACTH, adrenocorticotropic hormone; COR, cortisol; DST, overnight dexamethasone suppression test; LDDST, Low-dose dexamethasone suppression test; —: No data available for this item.

**Figure 1:**
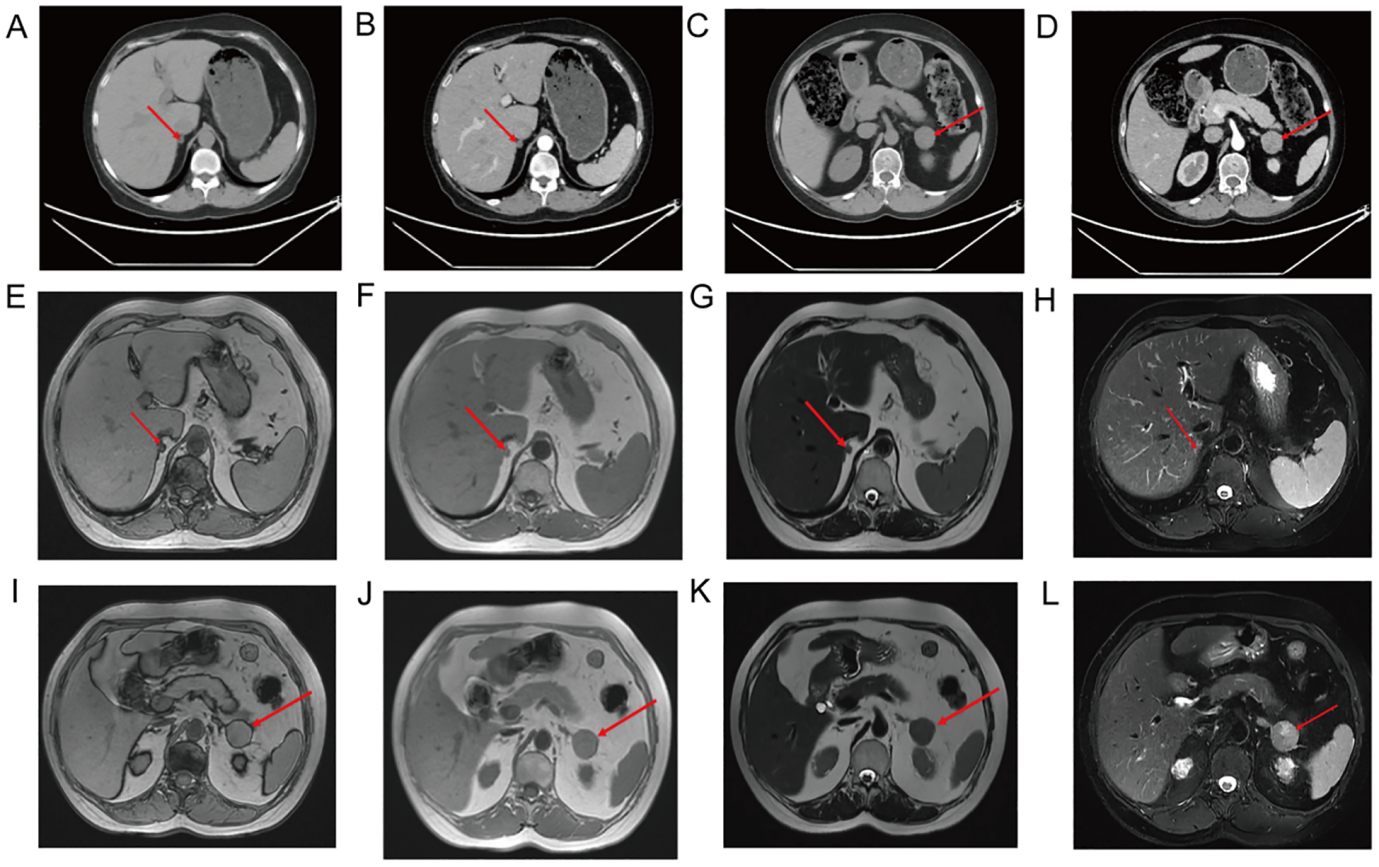
Multimodal imaging of the adrenal glands in Case 1. **(A)** Unenhanced CT demonstrating an 8 mm hypodense nodule in the right adrenal gland, with an attenuation value of −9 HU (arrow). **(B)** Contrast-enhanced CT demonstrating an 8 mm hypodense nodule in the right adrenal gland (arrow). **(C)** Unenhanced CT demonstrating an 28×27 mm hypodense nodule in the left adrenal gland, with an attenuation value of 26 HU (arrow). **(D)** Contrast-enhanced CT demonstrating an 28×27 mm hypodense nodule in the left adrenal gland (arrow). **(E)** T1-weighted out-of-phase MRI of the right adrenal gland. **(F)** T1-weighted in-phase MRI of the right adrenal gland. **(G)** T2-weighted MRI of the right adrenal gland. **(H)** Fat-suppressed T2-weighted MRI of the right adrenal gland. **(I)** T1-weighted out-of-phase MRI of the left adrenal gland. **(J)** T1-weighted in-phase MRI of the left adrenal gland. **(K)** T2-weighted MRI of the left adrenal gland. **(L)** Fat-suppressed T2-weighted MRI of the left adrenal gland. CT, computed tomography; MRI, magnetic resonance imaging; HU, hounsfield unit.

Cross-sectional imaging characterized bilateral adrenal lesions. Unenhanced and contrast-enhanced adrenal CT showed a small, indeterminate hypodense nodule (approximately 8 mm) in the right adrenal body and a larger, well-defined isodense mass (28 × 27 mm) in the left adrenal region. T1-weighted, Fat-suppressed T2-weighted adrenal magnetic resonance imaging (MRI) demonstrated avid enhancement of right adrenal body (approximately 7 mm) heterogeneous enhancement of a left-sided lesion (approximately 25 × 25 mm) involving the lateral limb of the adrenal gland (**Figure 1**).

In light of the discordant biochemical phenotype–characterized by ACTH-independent CS and an elevated ARR with non-suppressed post-saline ALD–AVS was performed to lateralize hormone excess. Following written informed consent, bilateral AVS was conducted, and plasma samples were analyzed by LC-MS/MS to map the adrenal steroid metabolome (**Supplementary Table 1**). Using 17OHP, ASD, DHEA as the reference hormones, successful bilateral cannulation was confirmed by the selectivity index. AVS results demonstrated left-sided COR hypersecretion and right-sided ALD hypersecretion (**Table 3**), establishing the diagnoses of unilateral PA and ACTH-independent CS.

**Table 3:** Lateralization of hormone secretion by AVS (Non-ACTH stimulation, synchronous blood sampling).

Case	Position	Result						
			ALD			COR		
		SI	LI_1_	LI_2_	CI	LI_1_	LI_2_	CI
		17-OHP correction						
case1	L_1_	10.32	0.22	0.15	4.05	9.12	4.17	0.66
	L_2_	18.26	0.22	0.15	4.05	7.48	3.42	0.54
	R_1_	32.92	4.62	4.62	18.74	0.11	0.13	0.07
	R_2_	9.25	6.49	6.49	26.32	0.24	0.29	0.16
case2	L_1_	8.5	3.2	—	0.26	0.67	—	0.42
	L_2_	11.75	16.15	—	1.29	0.64	—	0.4
	R_1_	22.67	0.31	0.06	0.08	1.48	1.56	0.62
	R_2_	0.25	—	—	—	—	—	—
		ASD correction						
case1	L_1_	7.25	0.08	0.09	5.8	3.22	2.51	0.94
	L_2_	127.89	0.08	0.1	6.5	2.92	2.27	0.85
	R_1_	8.22	13.24	11.82	76.8	0.31	0.34	0.29
	R_2_	3.9	10.95	9.79	63.5	0.4	0.44	0.38
case2	L_1_	20.6	1.31	—	0.11	0.28	—	0.17
	L_2_	32.23	5.88	—	0.47	0.24	—	0.14
	R_1_	22.93	0.76	0.17	0.08	3.55	4.23	0.61
	R_2_	0.27	—	—	—	—	—	—
		DHEA correction						
case1	L_1_	5.89	0.67	0.64	7.06	27.9	17.38	1.16
	L_2_	11.48	0.62	0.56	6.5	20.77	12.94	0.86
	R_1_	57.79	1.5	1.62	10.56	0.04	0.05	0.04
	R_2_	21.96	1.57	1.71	11.11	0.06	0.08	0.07
case2	L_1_	9.95	1.12	—	0.22	0.24	—	0.36
	L_2_	16.45	4.76	—	0.92	0.8	—	0.28
	R_1_	9.5	0.89	0.21	0.19	4.14	5.21	1.48
	R_2_	1.5	—	—	—	—	—	—

ACTH, adrenocorticotropic hormone; COR, cortisol; ALD, aldosterone; ASD, androstenedione; DHEA, dehydroepiandrosterone; 17OHP, 17α-hydroxyprogesterone; SI: Ratio of adrenal vein to inferior vena cava 17OHP (or ASD or DHEA). LI: Ratio of dominant-side ALD (or COR)/17OHP (or ASD or DHEA) to non-dominant-side ALD (or COR)/17OHP (or ASD or DHEA). CI: Ratio of non-dominant-side ALD (or COR)/17OHP (or ASD or DHEA) to inferior vena cava ALD (or COR)/17OHP (or ASD or DHEA). —: No data available for this item. After normalization using three different hormones—17-OHP, ASD, and DHEA—Case 1 confirmed successful bilateral cannulation, with the left side showing COR hypersecretion and the right side demonstrating ALD hypersecretion. In Case 2, prompts successful blood collection on the left side and R1, while blood collection on R2 fails. However, the right side exhibited COR hypersecretion, and the left side showed ALD hypersecretion. Both cases demonstrated clear lateralization.

**Figure 2:**
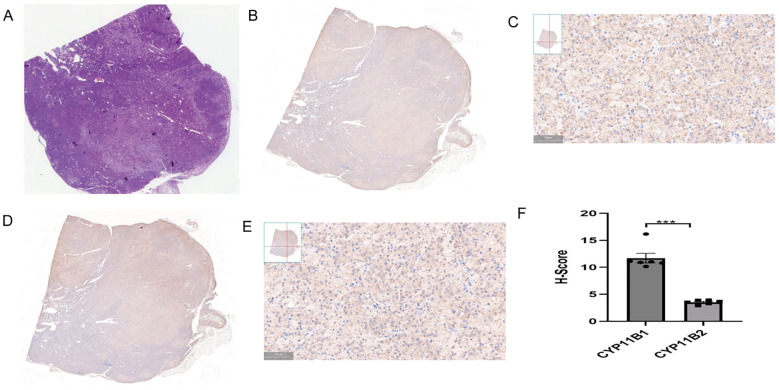
Pathological and immunohistochemical characterization of adrenocortical adenomas in case 1. **(A)** Representative H&E-stained section of an adrenal cortical adenoma demonstrating a typical cord-like and nested architectural pattern. Scale bar = 2500 μm, 4× magnification. **(B)** Gross morphological appearance of the resected adrenal specimen. Scale bar = 2000 μm, 6.5× magnification. **(C)** Immunohistochemical staining for CYP11B1 (11β-hydroxylase) showing strong positive immunoreactivity within the adenoma. Scale bar = 100 μm, 200× magnification. **(D)** Gross morphological appearance of the resected adrenal specimen. Scale bar = 2000 μm, 6.5× magnification. **(E)** Immunohistochemical staining for CYP11B2 (ALD synthase) demonstrating weak positive immunoreactivity. Scale bar = 100 μm, 200× magnification. **(F)** Semiquantitative analysis of CYP11B1 and CYP11B2 expression levels assessed by H-score. Statistical significance was determined using the Mann–Whitney U test (p < 0.001). H&E, hematoxylin and eosin; ALD, aldosterone; CYP11B1, 11β-hydroxylase; CYP11B2, aldosterone synthase.

The patient subsequently underwent laparoscopic left adrenalectomy targeting the CPA. Histopathologic examination confirmed a CPA (**Figure 2**). Immunohistochemistry (IHC) demonstrated strong positivity for CYP11B1 (11β-hydroxylase) and weak expression of CYP11B2 (ALD synthase) (**Figure 2**). One week postoperatively, the serum COR nadir was 1.43 µg/dL, consistent with surgical remission. Perioperative stress-dose glucocorticoids were administered to prevent adrenal crisis, and prednisone was gradually tapered over 14 months based on clinical and biochemical parameters. The patient remains on maintenance therapy with spironolactone (20 mg twice daily) and metformin (500 mg twice daily).

Her blood pressure was well controlled at 100–110/60–80 mmHg, and serum potassium remained within normal limits. Postoperative laboratory evaluation revealed the following: DRC 24.49 pg/mL, ALD 358.59 pg/mL, ARR 14.64, ACTH 31.11pg/mL (8:00 AM), COR 12.43 μg/dL (8:00 AM), and HbA1c 6.5%.

### 2.2 Case 2

A 60-year-old postmenopausal woman was referred to our endocrinology department for evaluation of incidentally discovered bilateral adrenal masses. Five years prior, she had been diagnosed with grade 3 HTN (peak 190/110 mmHg) and was managed with amlodipine besylate 10 mg daily, achieving suboptimal control (120–145/90–100 mmHg). Three months before admission, she developed unexplained fatigue and peripheral numbness. Abdominal CT demonstrated bilateral adrenal space-occupying lesions, and the patient was admitted for evaluation of the hormonal secretory profile and benign-malignant nature of the lesions. Her past medical history included endoscopic polypectomy for gastric polyps one year prior. Family history was notable only for HTN in her father; there was no reported family history of diabetes. She reached menopause at age 49 and had a history of full-term pregnancies.

On admission, vital signs were stable with a blood pressure of 121/84 mmHg and a regular pulse of 73 bpm. Anthropometric measurements revealed a BMI of 22.43 kg/m², waist circumference of 87 cm, and a WHR of 0.97, indicating central adiposity. Notably, there were no clinical stigmata of hypercortisolism, including centripetal obesity, moon facies, buffalo hump, or violaceous striae. Cardiopulmonary and abdominal examinations were otherwise unremarkable.

**Figure 3:**
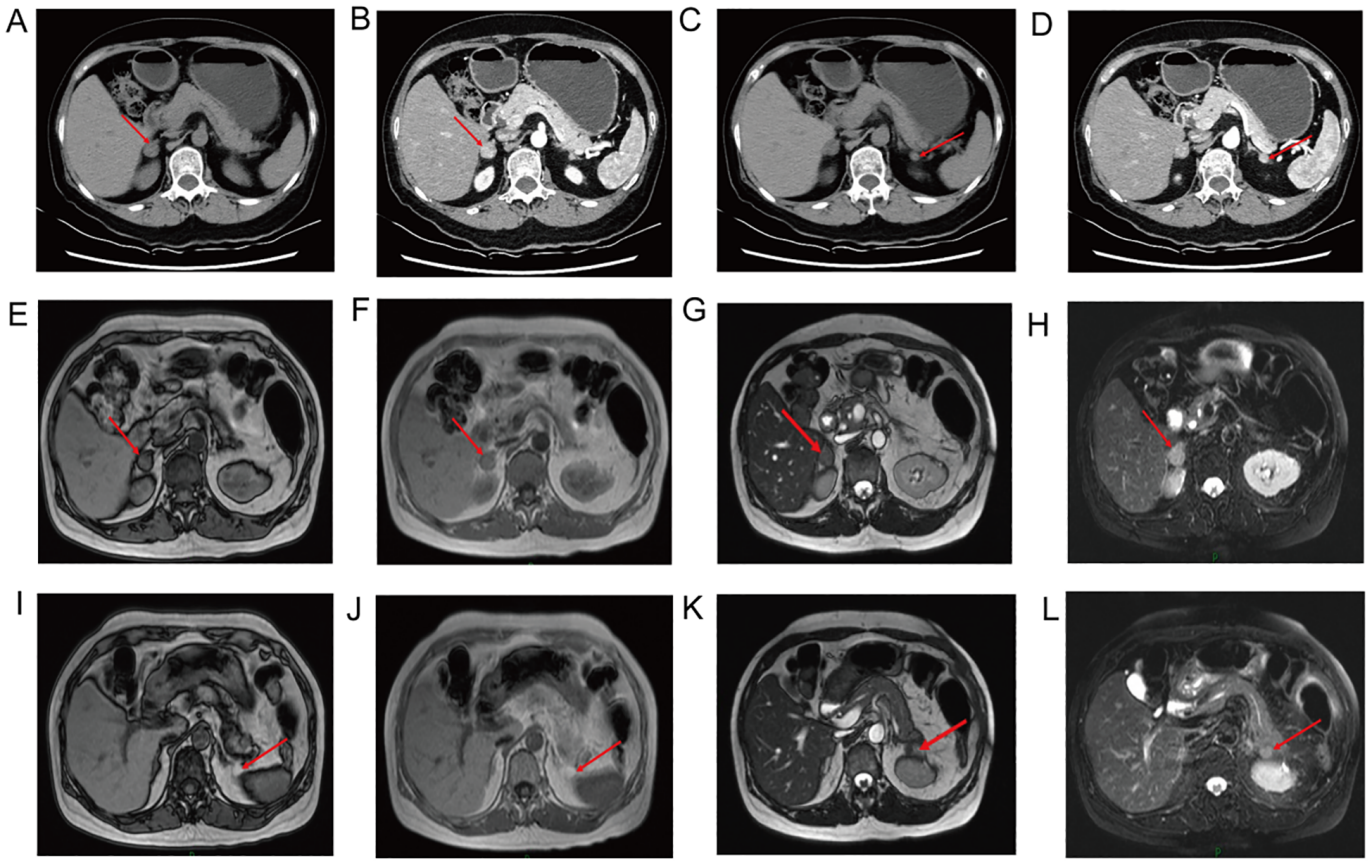
Multimodal imaging of the adrenal glands in Case 2. **(A)** Unenhanced CT demonstrating an19×18mm hypodense nodule in the right adrenal gland, with an attenuation value of 22 HU (arrow). **(B)** Contrast-enhanced CT demonstrating an19×18mm hypodense nodule in the right adrenal gland. **(C)** Unenhanced CT demonstrating an 10x11 mm hypodense nodule in the left adrenal gland, with an attenuation value of 9 HU (arrow). **(D)** Contrast-enhanced CT demonstrating an 10x11 mm hypodense nodule in the left adrenal gland (arrow). **(E)** T1-weighted out-of-phase MRI of the right adrenal gland. **(F)** T1-weighted in-phase MRI of the right adrenal gland. **(G)** T2-weighted MRI of the right adrenal gland. **(H)** Fat-suppressed T2-weighted MRI of the right adrenal gland. **(I)** T1-weighted out-of-phase MRI of the left adrenal gland. **(J)** T1-weighted in-phase MRI of the left adrenal gland. **(K)** T2-weighted MRI of the right adrenal gland. **(L)**. Fat-suppressed T2-weighted MRI of the left adrenal gland. CT, computed tomography; MRI, magnetic resonance imaging; HU, hounsfield unit.

Baseline laboratory evaluation revealed an HbA1c of 6.1% and normal glucose tolerance on OGTT (2-hour postload glucose 6.6 mmol/L). Fasting lipid indices were unremarkable. Serum potassium was 4.13 mmol/L, with urinary potassium excretion of 64.88 mmol/24 h. Endocrine workup demonstrated an elevated upright ARR of 80.59 (plasma ALD 194.22 pg/mL; DRC 2.41 pg/mL). Saline infusion testing (**Table 1**) did confirm PA, as post-infusion ALD failed to suppress below the diagnostic threshold. Evaluation of glucocorticoid excess revealed loss of the COR circadian rhythm (**Table 2**), supporting the diagnosis of ACTH-independent CS. Plasma-free and urinary metanephrines were within normal limits. Cross-sectional imaging characterized bilateral adrenal lesions. CT revealed hypodense lesions measuring 10 × 11 mm (left) and 19 × 18 mm (right). T1-weighted, Fat-suppressed T2-weighted adrenal MRI corroborated these findings, with lesion dimensions of 10 × 9 mm (left) and 22 × 15 mm (right) (**Figure 3**). Given the discordant biochemical phenotype, AVS was performed following written informed consent. LC-MS/MS steroid profiling using 17-OHP, ASD and DHEA as reference hormones (**Supplementary Table 1**) demonstrated left-sided ALD hypersecretion and right-sided COR hypersecretion (**Table 3**), establishing the diagnoses of lateralized PA and ACTH-independent CS.

**Figure 4:**
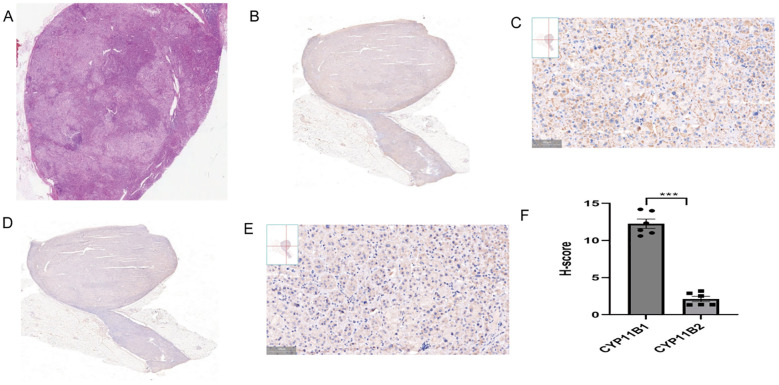
Pathological and immunohistochemical characterization of adrenocortical adenomas in case 2. **(A)** Representative H&E-stained section of an adrenal cortical adenoma demonstrating a typical cord-like and nested architectural pattern. Scale bar = 2500 μm, 4× magnification. **(B)** Gross morphological appearance of the resected adrenal specimen. Scale bar = 2000 μm, 6.5× magnification. **(C)** Immunohistochemical staining for CYP11B1 (11β-hydroxylase) showing strong positive immunoreactivity within the adenoma. Scale bar = 100 μm, 200× magnification. **(D)** Gross morphological appearance of the resected adrenal specimen. Scale bar = 2000 μm, 6.5× magnification. **(E)** Immunohistochemical staining for CYP11B2 (ALD synthase) demonstrating weak positive immunoreactivity. Scale bar = 100 μm, 200× magnification. **(F)** Semiquantitative analysis of CYP11B1 and CYP11B2 expression levels assessed by H-score. Statistical significance was determined using the Mann–Whitney U test (p < 0.001). H&E, hematoxylin and eosin; ALD, aldosterone.

Due to severe intraoperative adhesions between the tumor and the surrounding tissues, the patient underwent laparoscopic right adrenalectomy targeting the CPA. Histopathology confirmed an adrenocortical adenoma; IHC demonstrated strong positivity for CYP11B1 and weak focal positivity for CYP11B2 (**Figure 4**). One week postoperatively, the serum COR nadir was 1.96 µg/dL, indicating surgical remission. Perioperative stress-dose glucocorticoids prevented adrenal crisis, and prednisone was gradually tapered over 10 months. The patient is currently managed with spironolactone (20 mg twice daily).

Her blood pressure well controlled at 110–120/70–80 mmHg. Repeat laboratory evaluation showed HbA1c 5.8%, normal serum potassium, DRC 35.09 pg/mL, ALD 420.13 pg/mL, ARR 11.97, ACTH 22.02 pg/mL (8:00 AM), and COR 13.26 μg/dL (8:00 AM). The patient’s fatigue resolved completely without recurrence.

## 3 Discussion

The main novelty of the present report lies not merely in the coexistence of PA and ACTH-independent COR excess, but in the identification of bilateral adrenal adenomas with functionally opposing hormone secretion. Unlike typical A/CPAs, in which ALD and COR excess usually arise from a single adrenal lesion, our cases showed a clear contralateral pattern: one adrenal gland was predominantly responsible for COR excess, whereas the opposite gland was predominantly responsible for ALD excess. This distinctive phenotype represents the central feature of our report. Most previously described A/CPAs have been unilateral lesions with dual hormone secretion, whereas bilateral or multiple lesions are uncommon (12–14). Only a few reports have described functional dissociation between the adrenal glands. Lee et al. reported bilateral adrenocortical masses independently producing ALD and COR (15); Zhang et al. described a patient with a left COR-producing tumor and a right ALD-secreting tumor (16); and Ren et al. reported bilateral adrenal adenomas with left-sided COR and right-sided ALD hypersecretion (17). Extending these observations, our two cases demonstrated a reciprocal contralateral secretion pattern: Case 1 showed left-sided COR dominance and right-sided ALD dominance, whereas Case 2 showed right-sided COR dominance and left-sided ALD dominance. This opposing functional arrangement distinguishes our cases from more typical unilateral A/CPA, in which ALD and COR excess usually arise from the same side and COR excess is often mild. Compared with similar cases reported in the literature, our cases are distinctive in three respects. First, AVS was performed with LC-MS/MS–based steroid profiling using 17-OHP, ASD, and DHEA as non-COR reference hormones. Second, rather than proceeding to bilateral adrenal tumor resection, we adopted an adrenal-sparing strategy by laparoscopically resecting only the COR-dominant lesion, while managing residual ALD excess medically. Third, although Case 1 showed markedly higher midnight COR and post-1-mg dexamethasone suppression test COR levels than Case 2, both patients exhibited features consistent with mild autonomous cortisol secretion (MACS) rather than overt CS. This dissociation between biochemical and clinical findings may be explained by two possible factors. First, hypercortisolism may have been identified at a relatively early stage, before cumulative end-organ effects were sufficient to produce overt Cushingoid features. Instead, COR excess initially manifested as cardiometabolic abnormalities, consistent with the clinical spectrum of MACS. Second, although not directly assessed in our patients, differences in tissue glucocorticoid sensitivity—potentially related to glucocorticoid receptor polymorphisms or local regulation by 11β-hydroxysteroid dehydrogenase enzymes—may also contribute to this discordance (18, 19). Although both patients exhibited mild clinical phenotypes, Case 1 demonstrated greater biochemical COR excess and more profound hypothalamic–pituitary–adrenal axis (HPA-axis) suppression than Case 2, which was reflected by a longer duration of postoperative glucocorticoid replacement. These findings suggest that preoperative COR burden and the degree of ACTH suppression may be more informative than overt Cushingoid features for predicting postoperative adrenal insufficiency and HPA-axis recovery.

This opposing secretion pattern has important clinical implications, as patients with A/CPAs have been reported to show a higher prevalence of metabolic disorders and cardiovascular complications than those with pure PA (20, 21). Moreover, autonomous COR co-secretion may interfere with COR-corrected AVS indices, potentially leading to inaccurate lateralization, inappropriate surgical decision-making, and postoperative adrenal insufficiency (13). A previous study reviewed six cases of bilateral adrenal adenomas with functionally distinct hormone secretion between the two adrenal glands. Most patients were female, aged 30–55 years, and all had HTN, with COR excess mainly presenting as MACS (22). The clinical profiles of our two patients were largely consistent with those reported in this series. Consistent with these observations, both patients in our series had long-standing, difficult-to-control HTN before the diagnosis; one had overt hypokalemia, whereas the other presented with fatigue. These findings also align with previous evidence suggesting that older age, longer HTN duration, and larger tumor diameter may be associated with A/CPAs (23).

In contrast to typical unilateral A/CPA, the present cases posed an additional challenge because the dominant sources of ALD and COR excess were located in opposite adrenal glands. Cross-sectional imaging identified bilateral adrenal lesions but could not determine the functional contribution of each lesion, making accurate lateralization essential for treatment planning. In conventional AVS for PA, ALD is usually normalized to COR to account for sample dilution and to confirm catheter selectivity, and lateralization is assessed by comparing ALD-to-COR ratios between the two adrenal veins (1, 24). However, when COR secretion is autonomous and asymmetric, COR can no longer be considered a neutral reference hormone (22). COR co-secretion may distort selectivity and lateralization indices during AVS, thereby increasing the risk of inaccurate lateralization and inappropriate surgical decision-making (25). In addition, unrecognized autonomous COR secretion may suppress the hypothalamic–pituitary–adrenal axis and increase the risk of postoperative adrenal insufficiency after adrenalectomy.

Mechanistically, unilateral autonomous COR secretion may suppress COR production from the contralateral adrenal gland, thereby compromising conventional COR-based AVS interpretation. COR excess from the COR-dominant side may overestimate selectivity, whereas contralateral COR suppression may artificially amplify ALD-to-COR ratios on the ALD-dominant side. To overcome this limitation, we employed LC-MS/MS-based steroid profiling using 17-OHP, ASD, and DHEA as non-cortisol reference hormones (26). This approach is supported by accumulating evidence indicating that these steroids may serve as superior correction factors; Notably, SIs calculated using DHEA-S or ASD have been shown to be 2- to 3-fold higher than COR-based SIs, and Ceolotto et al. reported that 17-OHP- and ASD-based SIs were 1.6-fold and 12.0-fold higher, respectively (27, 28). In our two cases, lateralization indices were highly consistent across all three non-cortisol markers, confirming contralateral hormone dominance: Case 1 exhibited left-sided COR dominance and right-sided ALD dominance, whereas Case 2 demonstrated the inverse pattern. These findings highlight the clinical value of multi-steroid AVS in the functional assessment and individualized management of bilateral adrenal adenomas with opposing ALD and COR dominance.

From a therapeutic perspective, the opposing secretion pattern also had direct implications for management. Resection of the ALD-dominant lesion alone would not have corrected clinically relevant COR excess, whereas bilateral adrenalectomy would have exposed the patients to lifelong adrenal insufficiency. Therefore, rather than removing both adrenal lesions, we adopted an adrenal-sparing strategy guided by multi-steroid AVS. Both patients underwent laparoscopic resection of the COR-dominant side, followed by postoperative declines in COR levels consistent with remission of hypercortisolism. Pathological evaluation confirmed adrenocortical adenomas with a CYP11B1-dominant steroidogenic phenotype.

For residual ALD excess, additional management options were carefully considered, including further surgery and medical therapy. After excluding clinically significant adrenal insufficiency and considering treatment efficacy, cost, and patient preference, mineralocorticoid receptor antagonist therapy was selected. This strategy achieved sustained biochemical control and symptomatic improvement. Together, these cases highlight the value of precise functional localization in guiding individualized, adrenal-sparing treatment decisions for patients with bilateral adrenal adenomas and opposing ALD/COR dominance.

## 4 Conclusion

These cases illustrate a rare bilateral adrenal phenotype with opposing ALD and COR dominance. Multi-steroid AVS overcame the limitations of imaging and COR-based AVS, enabling accurate lateralization, targeted resection of the cortisol-dominant lesion, and medical control of residual ALD excess. Recognition of this pattern is essential for individualized adrenal-sparing management and for avoiding inappropriate adrenalectomy or postoperative adrenal insufficiency. Larger case series are warranted to further characterize the clinical spectrum and elucidate the pathogenesis of this distinct subset of A/CPAs.

## Data Availability

The original contributions presented in the study are included in the article/**Supplementary Material**. Further inquiries can be directed to the corresponding author.
