# Supplementary material for: Bilateral adrenal adenomas presenting with ACTH-independent Cushing’s syndrome and primary aldosteronism: diagnostic insights from multi-steroid adrenal venous sampling
Source: Front Endocrinol (Lausanne). 2026 Aug 3;17:1861198. doi: 10.3389/fendo.2026.1861198 (PMC13477915; doi:10.3389/fendo.2026.1861198)
Supplement: Supplementary file 1 [file Table1.docx]

| Hormone (unit) | Reference range | Case1 | | | | | Case2 | | | | |
| --- | --- | --- | --- | --- | --- | --- | --- | --- | --- | --- | --- |
|  |  | V | L_1_ | L_2_ | R_1_ | R_2_ | V | L_1_ | L_2_ | R_1_ | R_2_ |
| Testosterone (ng/mL) | 0.02–0.45 | 0.07 | 0.3 | 0.52 | 0.4 | 0.27 | 0.1 | 0.17 | 0.2 | 0.19 | 0.04 |
| Dihydrotestosterone (ng/mL) | ≤0.30 | 0.01 | 0.02 | 0.02 | 0.03 | 0.02 | 0.14 | 0.13 | 0.15 | 0.36 | 0.13 |
| Androstenedione (ng/mL) | 0.20–0.75 | 0.99 | 7.18 | 11.51 | 8.14 | 3.86 | 0.3 | 6.18 | 9.67 | 6.88 | 0.08 |
| Dehydroepiandrosterone (ng/mL) | ＜8.0 | 0.56 | 3.3 | 6.43 | 32.36 | 12.30 | 0.20 | 1.99 | 3.29 | 1.90 | 0.30 |
| Dehydroepiandrosterone sulfate (ng/mL) | 270–2400 | 392.73 | 387.48 | 411.2 | 600.57 | 589.29 | 163 | 176 | 189 | 174 | 134 |
| 17-Hydroxyprogesterone (ng/mL) | ＜0.51 | 0.53 | 5.47 | 9.68 | 17.54 | 4.90 | 0.24 | 2.04 | 2.82 | 5.44 | 0.06 |
| Progesterone (ng/mL) | ≤0.2 | ＜0.10 | 1.04 | 1.46 | 2.22 | 0.86 | 0.30 | 0.30 | 0.40 | 1.10 | 0.40 |
| Cortisol (ng/mL) | 60–260 | 160.54 | 1096.43 | 1590.32 | 385.43 | 235.23 | 92 | 326 | 429 | 1290 | 79 |
| 11-deoxycortisol (ng/mL) | ≤1.07 | 1.12 | 9.59 | 10.75 | 9.59 | 6.17 | 1.12 | 8.73 | 12.43 | 47.26 | 0.35 |
| Cortisone (ng/mL) | 12–35 | 26.94 | 93.65 | 121.42 | 23.47 | 22.28 | 27 | 27 | 36 | 71 | 1 |
| Corticosterone (ng/mL) | 0.53–15.6 | 4.92 | 67.37 | 100.80 | 28.23 | 13.33 | 2.74 | 20.66 | 29.22 | 121.79 | 1.82 |
| 17-Hydroxypregnenolone (ng/mL) | 0.31–4.55 | 0.33 | 7.15 | 11.94 | 35.36 | 11.93 | 1.33 | 9.11 | 6.48 | 7.13 | 3.43 |
| Estrone (pg/mL) | ≤0.065 | 129.75 | 75.59 | 48.6 | 63.87 | 54.64 | 0.025 | 0.013 | 0.038 | 0.012 | 0.015 |
| Estradiol (pg/mL) | ＜0.055 | 222.25 | 43.45 | 32.81 | 60.17 | 41.64 | 0.01 | 0.016 | 0.011 | 0.012 | 0.01 |
| 11-deoxycorticosterone (ng/mL) | ≤0.18 | 0.12 | 2.73 | 4.04↑ | 3.62 | 1.37 | 0.11 | 0.35 | 0.50 | 1.06 | 0.04 |
| Aldosterone (ng/mL) | 0.03–0.16 | 0.01 | 0.42 | 0.75 | 6.26 | 2.45 | 0.06 | 0.13 | 0.91 | 0.11 | 0.16 |

Supplementary table 1. AVS steroid hormone profile (Non-ACTH stimulation, synchronous blood sampling). AVS:Adrenal Vein Sampling
